# Multiclass imbalance learning: Improving classification of pediatric brain tumors from magnetic resonance spectroscopy

**DOI:** 10.1002/mrm.26318

**Published:** 2016-07-12

**Authors:** Niloufar Zarinabad, Martin Wilson, Simrandip K Gill, Karen A Manias, Nigel P Davies, Andrew C Peet

**Affiliations:** ^1^ Institute of Cancer and Genomic SciencesUniversity of BirminghamBirminghamUnited Kingdom; ^2^ Birmingham Children's Hospital NHS Foundation TrustBirminghamUnited Kingdom; ^3^ School of Psychology and Birmingham University Imaging CentreUniversity of BirminghamEdgbastonBirmingham United Kingdom; ^4^ Department of Medical PhysicsUniversity Hospitals Birmingham NHS Foundation TrustBirminghamUnited Kingdom

**Keywords:** MR spectroscopy, imbalanced learning, brain tumors, diagnosis, classification

## Abstract

**Purpose:**

Classification of pediatric brain tumors from ^1^H‐magnetic resonance spectroscopy (MRS) can aid diagnosis and management of brain tumors. However, varied incidence of the different tumor types leads to imbalanced class sizes and introduces difficulties in classifying rare tumor groups. This study assessed different imbalanced multiclass learning techniques and compared the use of complete spectra and quantified metabolite profiles for classification of three main childhood brain tumor types.

**Methods:**

Single‐voxel, Short echo time MRS data were collected from 90 patients with pilocytic astrocytoma (n = 42), medulloblastoma (n = 38), or ependymoma (n = 10). Both spectra and metabolite profiles were used to develop the learning algorithms. The borderline synthetic minority oversampling technique and AdaboostM1 were used to correct for the skewed distribution. Classifiers were trained using five different pattern recognition algorithms.

**Results:**

Use of imbalanced learning techniques improved the balanced accuracy rate (BAR) of all classification methods (average BAR over all classification methods for spectra: oversampled data = 0.81, original = 0.63, *P* < 0.001; metabolite concentration: oversampled‐data = 0.91, original = 0.75, *P* < 0.0001). Performance of all classifiers in discriminating ependymomas increased when oversampled data were used compared with original data for both complete spectra (F‐measure *P* < 0.01) and metabolite profile (F‐measure *P* < 0.001).

**Conclusion:**

Imbalanced learning techniques improve the classification accuracy of childhood brain tumors from MRS where group sizes differ and facilitate the inclusion of rarer tumor types into clinical decision support systems. Magn Reson Med 77:2114–2124, 2017. © 2016 The Authors Magnetic Resonance in Medicine published by Wiley Periodicals, Inc. on behalf of International Society for Magnetic Resonance in Medicine. This is an open access article under the terms of the Creative Commons Attribution License, which permits use, distribution and reproduction in any medium, provided the original work is properly cited.

## INTRODUCTION

Brain tumors are the most common solid tumors in childhood. They comprise approximately 25% of all pediatric cancers and are the leading cause of cancer death in children 1. ^1^H‐magnetic resonance spectroscopy (MRS) has been used as a noninvasive diagnostic tool for a variety of pathologies 2, 3, 4 and there are number of studies on the significant contribution of MRS for the characterization of pediatric brain tumors 5, 6, 7, 8, 9. Pattern recognition based classification of brain tumors using MRS data has been investigated thoroughly for more than two decades now 6, 8, 10, 11 and its application for discriminating childhood brain tumors has been explored in single and multicenter studies. Previous studies have used both complete spectra and quantified metabolite profiles for creation of an optimum and objective decision support system demonstrating promising outcomes 11, 12, 13.

However, challenges remain in pediatric brain tumor classification using in vivo MRS data. The main issue is the limited number of available cases for rare tumor types. In a given classification task, the size of the data set has an important role in building reliable learning algorithms. An imbalanced multiclass data set makes uncovering regularities within the small rare tumor group (minority class) challenging and introduces difficulties in constructing accurate learning algorithms and robust conclusions 14, 15. This issue worsens in cases of dealing with multi‐majority/multi‐minority data sets. In grading and classifying childhood brain tumors, correcting for the skewed distribution of the data is crucial, because it is costly to misclassify the cases from rare tumor types.

In the past decade, with the growth in availability of data, the concept of learning from imbalanced data has attracted growing attention in many disciplines and has advanced significantly. Imbalanced learning techniques have been used for a wide range of applications such as fraud detection, finance, oil spill detection, network intrusion, and biomedical studies 16, 17, 18, 19, 20, 21. However, to the best of our knowledge, there is no previous work on classification of brain tumor MRS data considering the class imbalance issue. In previous brain tumor classification studies, either this issue is neglected or the study cohort has simply been selected to include only tumor groups with more than a specific number of cases.

In this study, we focused on the development of reliable imbalanced pattern recognition techniques for classification of imbalanced MRS pediatric tumor data comparing complete spectra and quantified metabolite profiles. The objective was to develop a classification algorithm that provides high accuracy for the less common tumor types (minority class) without jeopardizing the majority class (common tumor types). A wide range of machine learning methods have been developed previously at the data and algorithm level for classification of imbalanced multiclass data 14, 15, 17, 22. In the present study, the synthetic minority oversampling technique and Ensemble learning were chosen as data and algorithmic level methods, respectively, to deal with this issue.

Among the available ensemble learning approaches, AdaBoost 23 and random forests 24 have become very popular for their simplicity, robustness, and adaptability to multiclass imbalanced data. AdaBoost multiclass (AdaBoostM1) 25 constructs an ensemble of subsidiary classifiers by applying a given base learning algorithm (weak learner) to successive derived training sets that are formed by reweighting the original training set according to a set of weights maintained over the training set. Initially, the weights assigned to each training instance are set to be equal and in subsequent iterations; these weights are later adjusted so that the weight of the instances misclassified by the previously trained classifier is increased, whereas that of the correctly classified ones is decreased. The AdaBoost technique attempts to produce new classifiers that are able to better predict the ‘‘hard” instances for the previous ensemble members.

The other widely used ensemble technique is the random forest. This technique works by means of generating an ensemble of classification trees by applying the tree to different permutated training sets created from the data set, then combining the outputs from each tree to create the prediction of the ensemble classifier. The combination is often performed by voting for the most popular class.

In addition to ensemble techniques, balancing the classes at the data level can be performed by over‐ or undersampling of the data. However, widely used random oversampling with replacement does not help the classification and suffers from overfitting. Random undersampling also weakens the performance of the classifier for the majority class by removing useful information. Chawla et al. 26 proposed the use of a synthetic minority oversampling technique (SMOTE) which is based on creating synthetic class examples. In this study, an adaption of SMOTE called borderline SMOTE (bSMOTE) has been used to allow for building a larger decision region that contains nearby instances of the minority class 27.

It has been demonstrated that SMOTE integrated to an ensemble boosting method can improve prediction of the minority class without sacrificing performance of the classifier for the majority and overfitting the minority 28, 29. Here, we investigate the integration of bSMOTE and ensemble boosting methods for the classification of imbalanced data and evaluate application of the bSMOTE to pediatric brain tumor classification.

## METHODS

Patient data were collected retrospectively from children who underwent single‐voxel MRS between July 2003 and March 2015 during a routine MRI for a suspected brain tumor prior to treatment. The enrolled cohort consisted of 90 patients (female, n = 42; male, n = 48; age, 6.86 ± 4.22 y) with three different tumor types, including medulloblastoma (n = 38), pilocytic astrocytoma (n = 42), and ependymomas (n = 10, four of which were anaplastic) from all regions of the brain. All tumors had tissue available, and histopathological, clinical, and radiological features were used to form a diagnosis agreed by a multidisciplinary team. Approval was obtained from the research ethics committee and informed consent given by parents/guardians.

### Data Acquisition

MRS was performed on a 1.5T MR scanner (GE Excite, Siemens Symphony, or Siemens Avanto) after conventional MRI, which included T1‐weighted, T2‐weighted, and T1‐weighted postcontrast sequences. Single‐voxel MRS data were acquired using a standard protocol (point‐resolved spectroscopy (PRESS) TE = 30 ms, TR = 1500 ms, spectral resolution 1 Hz/point). Cubic voxels were used with either 2 cm or 1.5 cm side length and 128 or 256 repetitions were acquired, respectively. A water unsuppressed acquisition was also acquired as a concentration reference. Voxel placement was entirely within the tumor as delineated by the conventional MRI with the enhancing component maximized.

### MRS Processing and Quality Control

Raw spectroscopy data were processed using TARQUIN version 4.3.6 with a basis set including 19 different metabolites and nine lipid and macromolecular components 30. Frequency alignment, zero order phase correction, baseline correction, and water removal using HSVD methods were applied by TARQUIN. TARQUIN determines the chemical shift offset, phase, and baseline during the fitting process. It then zero fills the time domain data by factor of 2 (×2) and converts the time domain signal to spectral domain using Fourier transform. The obtained spectra are then resampled to 0.49 Hz/point. This is to ensure all cases have a consistent Hz/point. The resampled spectra are used for analysis in this study. The spectral range used for metabolite analysis and classification was set to 0.5 to 4 ppm to include the signals of interest.

All enrolled cases passed the following quality control criteria: signal‐to‐noise ratio ≥ 4; full‐width half‐maximum ≤ 0.15 ppm; stable baseline; good phasing; adequate water suppression; and absence of artifacts. We defined signal‐to‐noise ratio as the ratio between the maximum in the spectrum minus baseline divided by 2 × the root mean square of the spectral noise level.

The voxel was also reviewed to ensure it was positioned over the tumor, did not include significant amounts of normal‐appearing brain or cyst, and was at least 3 mm away from lipid‐containing bone and scalp.

For the quantified metabolite profiles, Cramer‐Rao lower bounds were calculated to evaluate metabolite accuracy. All metabolites where at least two patients had a Cramer‐Rao lower bound of <50 were included (aspartate, alanine, and gamma‐aminobutyric acid (GABA) were excluded from analysis) 8.

The following metabolites were used for classification: citrate, glycerophosphocholine, glucose, glutamine, glutathione, glutamate, glycine, myo‐inositol, lactate, phosphocholine, scyllo‐inositol, taurine, total creatine, and total *N*‐acetyl aspartate (NAA). The nine macromolecular and lipid components were grouped together (TLM) to account for three broad resonances at 0.9 ppm, 1.3 ppm, and 2.0 ppm, yielding a maximum of 17 variables.

### Classification

The ependymoma group accounted only for 11% of the total data and was thus considered the minority class. To correct for this imbalance in the data set, bSMOTE was used to overpopulate the ependymoma group.

The bSMOTE algorithm creates artificial data based on the feature space similarities between existing minority examples. To explain this technique, for each minority class sample ( $\;x_{i}\in S_{\text{min}}$), consider the K‐nearest neighbors for some specified integer *k* and call this set $S_{\text{iminNN}}$.

For each $x_{i}$, the numbers of samples in $S_{\text{iminNN}\;}$ that belong to the majority class are identified. Those $x_{i}$ with all neighbors from the majority class are excluded from the oversampling process. Those $x_{i}$ that have more majority class neighbors than minority class are selected to form the borderline minority set, $S_{\text{minBL}}$. These sets are most likely to be misclassified and are used for oversampling here. To create a synthetic sample, randomly select one of the K‐nearest neighbors of the $x_{i}\in S_{\text{minBL}}$, which is from $S_{\text{min}}$, then multiply the corresponding feature vector difference with a random number between [0,1]; finally, add this vector to $x_{i}$:

(1) $$x_{\text{new}}=x_{i}+\left({\hat{x}}_{i}-x_{i}\right)\times \delta$$

where $x_{i}\in S_{\text{minBL}}$ is the minority instance under consideration, ${\hat{x}}_{i}$ is one of the K‐nearest neighbors for $x_{i}$: ${\hat{x}}_{i}\in S_{\text{min}}$, and δ∈ [0,1] is a random number. Therefore, the resulting synthetic instance according to Equation 1 is a point along the line segment joining $x_{i}$ and the randomly selected K‐nearest neighbor ${\hat{x}}_{i}$.

**Figure 1:**
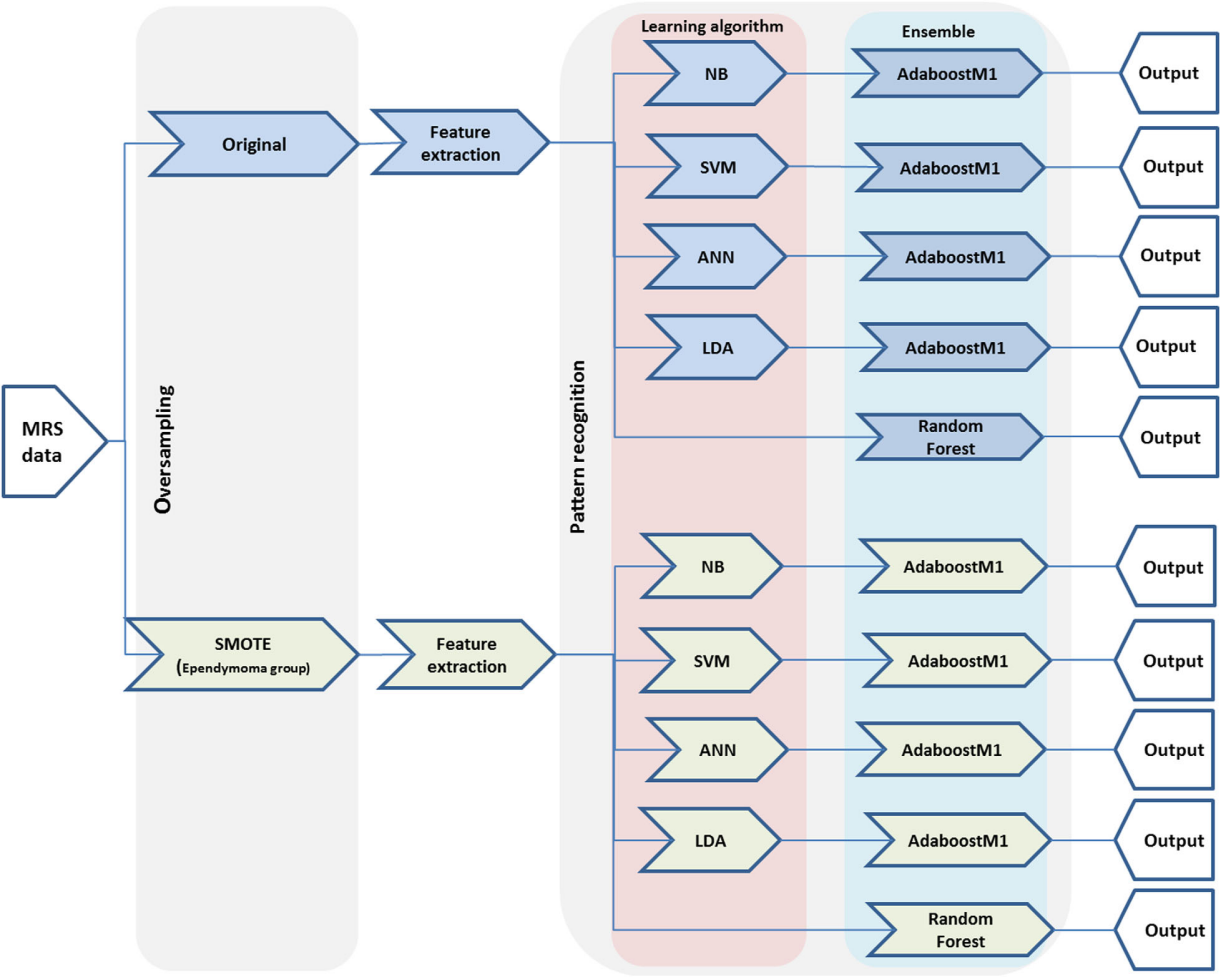
Experiment overview. These sets have been applied to both complete spectra and quantified metabolite profiles.

Oversampling was performed at 50%, 100%, 150%, and 200% (adding 5, 10, 15, and 20 synthetic samples respectively) using five nearest neighbors of each ependymoma to evaluate the oversampling size effects on the classification performance. Oversampling simulation at all rates was repeated 100 times and results were averaged to ensure robustness of bSMOTE. Performance of classifies trained using the original training data set and the training sets with overpopulated ependymoma (bSMOTE data set) for both complete spectra and metabolite profile are compared here (Fig. 1).

Principal component analysis was used for dimension reduction and extraction of features that best discriminate between the three tumor groups. Principal components accounting for 95% of variance were included.

To uncover interactions among metabolic and spectral features of the three tumor groups and illustrate the effect of class size on data discrimination, a linear projection method (FreeViz) based on the gradient optimization approach was used to produce a projection map 31. The projection map provided a global view of the imbalanced data classification problem. The FreeViz linear projection technique uses the interactions among attributes in different classes and finds a linear combination of features that best exhibits a trait specific structure of projected data (such as clusters) when it is mapped onto a two‐dimensional graph.

Classifiers were trained using random forests with an adaptive number of trees and AdaBoostM1 ensemble with four types of weak learners: Naive Bayes (NB), Support Vector Machine (SVM), artificial neural networks (ANN), and linear discriminative analysis (LDA). A radial basis (Gaussian) function kernel was chosen for SVM. In ANN, learning was performed by minimizing an L2‐regularized cost function. Three hidden layers were chosen with a sigmoid activation function for the nodes.

The AdaBoost ensemble technique was applied to the above classifiers and the outcome was compared with the random forest ensemble technique for both the original and bSMOTE data set.

Ten‐fold cross‐validation was used to evaluate the learning algorithm performance. To evaluate classifiers trained using bSMOTE data (as it contains synthetic instances) and ensure their stability and accuracy, the original data set was partitioned into *z* = 10 pools in which one pool is used for evaluation while the remaining *z* − 1 pools are added to the bSMOTE ependymoma cases, obtained from oversampling ependymoma cases in the remaining *z* − 1 pools, and used for training to produce mean error rates, where z ∈{1, 2, …, Z}. The pools were then randomly rotated and the subsampling and permutation tests were repeated until all pools had been evaluated exactly once. This ensured that there were no bSMOTE ependymoma cases in any of the test sets.

It should be noted that in the classification of imbalanced data, the commonly used accuracy measure reflects mainly the accuracy of classifying the majority group. Therefore, G‐mean and F‐measure 14, 15 metrics were introduced for more precise performance evaluation in class imbalance learning. G‐mean is considered most appropriate when the performance of all classes is concerned and is adapted from sensitivity and specificity measures:

$$G\;mean=\sqrt{sensitivity*specifity\;}.$$

G‐mean indicates the balance between classification performance of majority and minority classes. F‐measure is used when the performance of a single class (particularly the minority class) is considered important and is calculated via

$$F\;meaure=\frac{2*precison*sensitivity}{precision+sensitivity},$$

where precision (or the positive predictive value) is defined as the percentage of selected items that classify correctly (measure of exactness) and sensitivity (or in other terms recall) is the percentage of correctly classified items which are selected (measure of completeness). F‐measure incorporates both of above measures to express their trade‐off. The balanced accuracy rate of the learning algorithm (BAR) calculated as the mean of the accuracies for the three tumor types, is also reported here as a performance measure metric.

In this study, all learning algorithms were developed in Python (version 2.7) using Scikit‐learn (version 0.16.1) and Orange (version 2.6a2) libraries.

## RESULTS

The mean metabolite concentrations of the original three tumor groups are presented in Table 1. The Kruskal‐Wallis test for the analysis of variance (α = 0.05) was applied to determine the significant differences in metabolite concentrations between the three groups. Statistical analysis was performed using SPSS (version 21.0), and *P* < 0.05 was considered statistically significant.

**Table 1:** Estimated Metabolite Concentration ± Standard Deviation of the Three Tumour Types as Calculated by TARQUIN.

Metabolite	Ependymomas	Medulloblastoma	Pilocytic Astrocytoma	*P* a
Citrate	0.85 ± 0.48	0.51 ± 0.36	0.30 ± 0.30	<0.001
Creatine	2.04 ± 0.89	1.46 ± 1.34	0.73 ± 0.95	0.001
Glycerophosphocholine	1.56 ± 0.89	2.22 ± 1.16	0.77 ± 0.50	<0.001
Glucose	2.04 ± 1.23	1.6 ± 1.85	2.61 ± 1.83	0.046
Glutamine	2.80 ± 1.69	2.34 ± 1.97	3.20 ± 2.05	0.137
Glutathione	0.60 ± 0.71	0.54 ± 0.54	0.19 ± 0.38	0.003
Glutamate	3.94 ± 1.87	3.73 ± 2.73	2.03 ± 1.12	<0.001
Glycine	2.60 ± 4.20	3.21 ± 2.50	0.29 ± 0.60	<0.001
Myo‐inositol	6.19 ± 4.78	1.37 ± 3.52	1.23 ± 1.92	<0.001
Lactate	2.16 ± 1.64	2.30 ± 1.6	1.98 ± 1.05	0.557
NAA	0.24 ± 0.28	0.32 ± 0.40	0.56 ± 0.58	0.043
NAAG	0.70 ± 0.44	0.78 ± 0.58	0.95 ± 0.66	0.306
Phosphocholine	0.47 ± 0.40	1.14 ± 0.79	0.34 ± 0.33	<0.001
Phosphocreatine	2.12 ± 1.66	1.71 ± 1.19	0.48 ± 0.55	<0.001
Scyllo‐inositol	0.21 ± 0.30	0.32 ± 0.38	0.03 ± 0.12	<0.001
Taurine	1.58 ± 1.49	3.46 ± 2.50	0.66 ± 0.68	<0.001
Total NAA (NAA + NAAG)	0.94 ± 0.49	1.11 ± 0.77	1.50 ± 0.89	0.030
Total choline (glycerophosphocholine + phosphocholine)	2.03 ± 1.14	3.37 ± 1.59	1.12 ± 0.44	<0.001
Total creatine (creatine + phosphocreatine)	4.17 ± 1.89	3.18 ± 1.85	1.22 ± 1.29	<0.001
Glx (glutamine + glutamate)	6.75 ± 2.63	6.07 ± 3.32	5.27 ± 1.18	0.189
TLM09	6.11 ± 2.27	6.90 ± 4.12	3.91 ± 1.83	<0.001
TLM13	20.85 ± 16.4	19.88 ± 14.2	7.24 ± 5.0	<0.001
TLM20	8.04 ± 3.85	9.40 ± 3.89	5.0 ± 2.21	<0.001

Abbreviations: NAA, *N*‐acetyl aspartate; NAAG, N‐acetyl aspartate Glutamate; TLM, Total lipid and macromolecular.

Data are presented as the mean ± standard deviation.

a Calculated using Kruskal‐Wallis analysis of variance with α = 0.05 for ependymomas (n = 10) versus medullobastoma (n = 38) versus pilocytic astrocytoma (n = 42).

**Figure 2:**
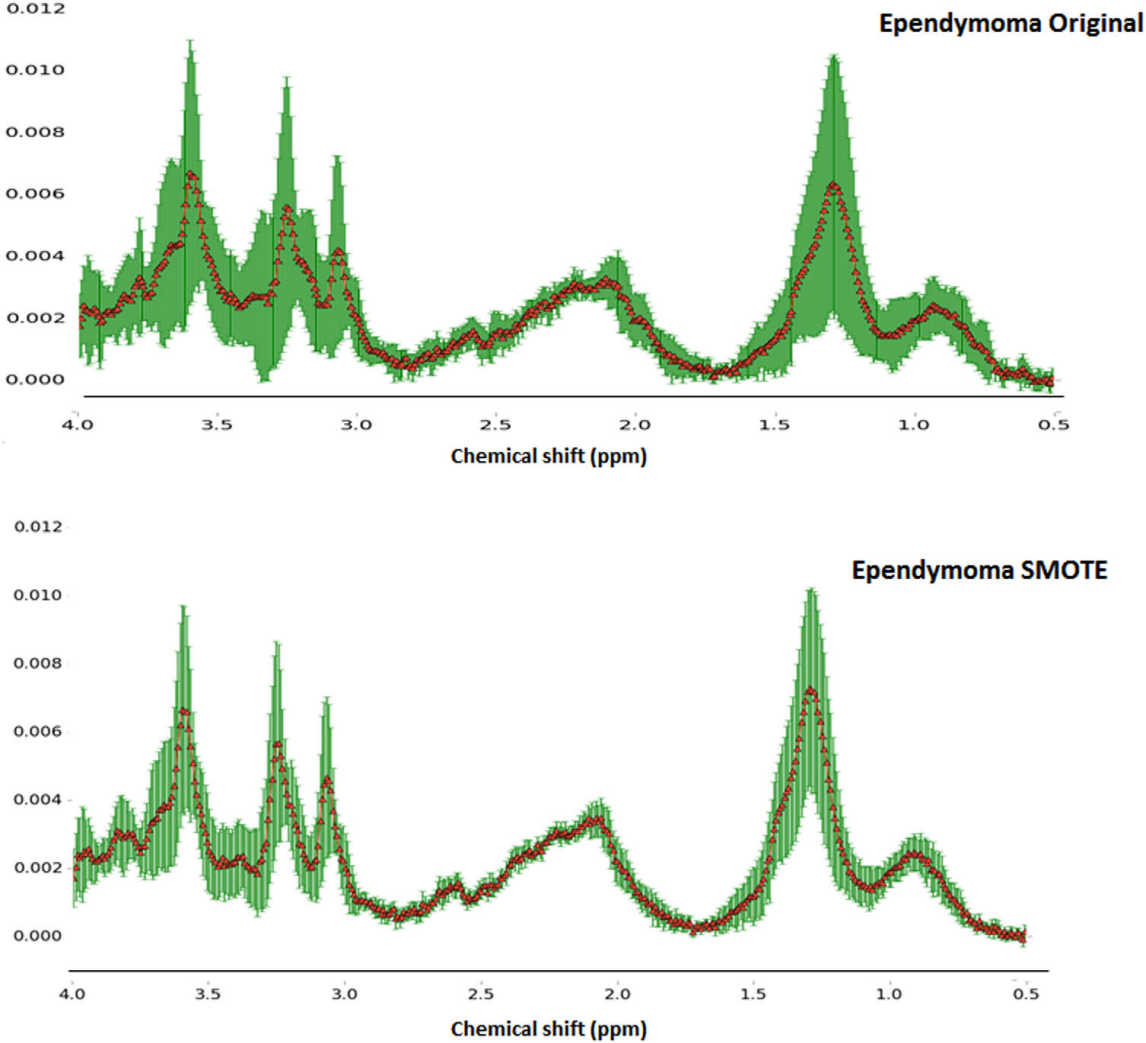
Mean ± standard deviation spectra for the original (n = 10) and synthetically generate ependymoma samples (n = 20) using bSMOTE and five nearest neighbors. Instances are created based on the original ependymoma (WHO grade II) and anaplastic ependymoma (World Health Organization grade III) samples.

The mean spectra of the 20 synthetic ependymoma cases generated using bSMOTE and the original ependymoma cases are illustrated in Figure 2. bSMOTE cases were created based on the original ependymoma (WHO grade II) and anaplastic ependymoma (WHO grade III) samples. Visual inspection of the bSMOTE mean spectra shows similarities with the key features of the true ependymoma spectra. The major peaks at 3.6, 3.2, and 3.0 ppm correspond well to the features of the ependymoma spectra.

The Kruskal‐Wallis test for the analysis of variance (α = 0.05) was applied to determine the significant differences in metabolite concentration between bSMOTE ependymoma and medulloblastoma, pilocytic astrocytoma, and the original ependymoma cohort. No significant difference was found between bSMOTE ependymoma and the original ependymoma group. Analysis of variance revealed significant differences between bSMOTE ependymoma, pilocytic astrocytoma, and medulloblastoma in 14 of the individual metabolites and combined macromolecules and lipids at 0.9, 1.3, and 2.0 ppm. Only lactate and glutamine had *P* values greater than 0.05. These differences reflect those seen with the original ependymoma data set and confirm that the bSMOTE ependymoma data set represents known ependymoma characteristics. bSMOTE ependymoma cases were added to the original dataset to increase the ependymoma group size by 50%, 100%, 150%, and 200% and evaluate the effect of minority class sample size on classification.

**Figure 3:**
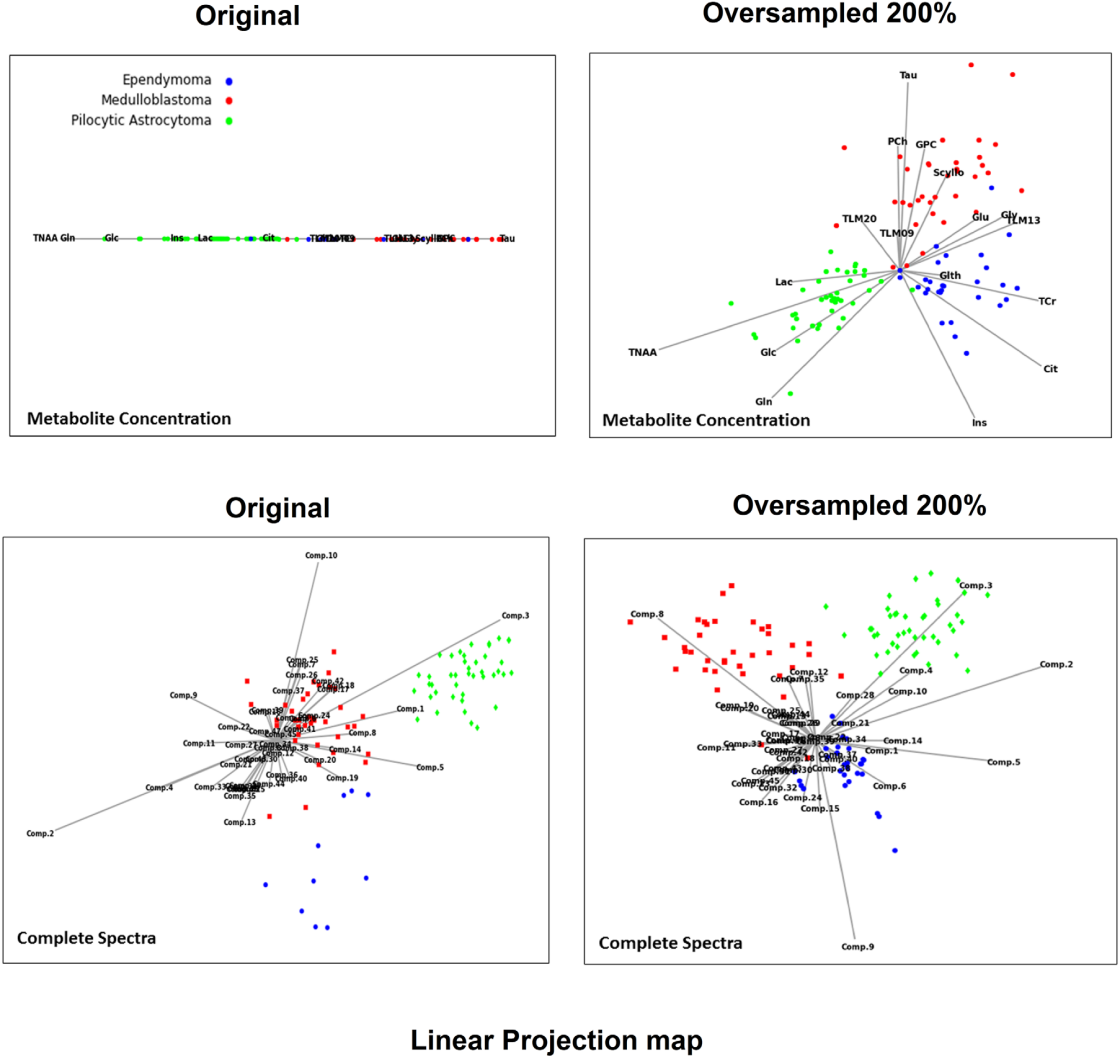
FreeViz two‐dimensional linear projection graphs visualizing the interactions among tumor groups in original and bSMOTE training data sets. Linear projection of the data provides new insight into the data through visualization of the space with reduced dimensionality. This method finds an optimal two‐dimensional linear projection of the given data, where the quality is defined by a separation of the data from different classes and the proximity of the instances from the same class. Base vectors of projection represent relevant metabolic features. Tumor types are identified by their color. Features with longer projection of the base vector are those with a higher impact on the placement of the instances in the two‐dimensional projection. Because this technique optimizes the projection with respect to classification of the groups, features with higher impact on classification outcome generally will have longer base vectors.

Figure 3 shows the projection maps of both the original metabolite profile and the complete spectra at a 200% oversampling rate. By increasing the number of ependymoma samples (minority class) and balancing the data set, a better separation of different tumor group cases was observed for both metabolite profile and complete spectra data sets. Studying the base feature vectors for metabolite profile data, taurine and total NAA were shown to be the key features for discriminating medulloblastoma and pilocytic astrocytoma, respectively. The impact of total creatine on classification of ependymoma and myo‐inositol in discriminating between pilocytic astrocytoma and ependymoma is apparent at a sampling rate of 200%. TLM13, glutamine, and glycine were shown to be the main features for separating ependymoma from medulloblastoma cases in the projection map when the oversampled data was used. Similarly, when the complete spectrum was used for the original data set, no base vector was observed that best separated the ependymoma group. Whereas at the 200% sampling rate, component nine was the feature with highest impact on discrimination of ependymoma cases, the ppm values that contributed the most to component nine were 2.58, 2.9, 3.05, 3.3 and 3.54.

For both complete spectra and metabolite profile, the original training data set and bSMOTE training sets were submitted to AdaBoostM1‐NB, AdaBoostM1‐SVM, AdaBoostM1‐ANN, AdaBoostM1‐LDA, and random forest classifiers, and their performance was compared. Principal component analysis was performed, and principal components accounting for 95% of variance were extracted, yielding 46 and 13 principal components for spectra and metabolite profiles, respectively.

The maximum number of weak learners assigned to all AdaboostM1 classifiers was set to 100 for metabolite profile and 200 for complete spectra. In case of perfect fit, the learning procedure was stopped early. For the random forest classifier, an increase in the number of ependymoma samples and therefore a higher number of trees were required to achieve optimal accuracy for both complete spectra and metabolite profile inputs (Table 2).

**Table 2:** Number of Trees used in Random Forest Classifier at Different Oversampling Rates.

bSMOTE %	0	50	100	150	200
Metabolite profile	25	25	50	70	90
Complete spectra	78	110	165	170	170

**Figure 4:**
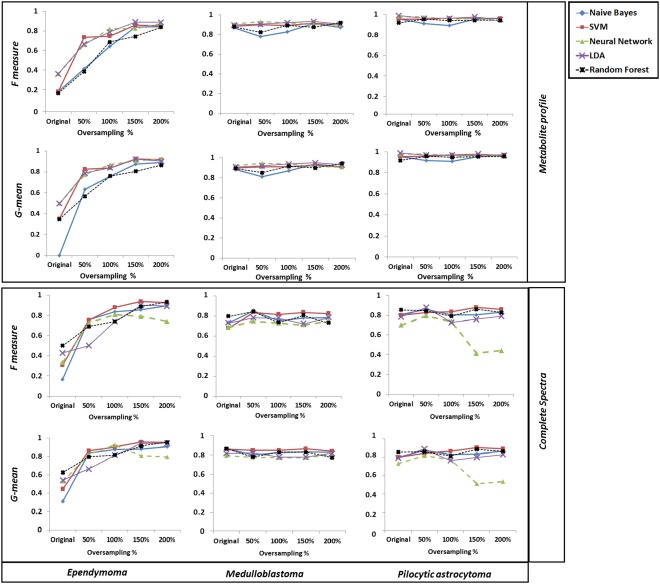
Performance comparison among five classification methods at different bSMOTE oversampling rates for complete spectra and metabolite profiles using the 10‐fold cross‐validation evaluation technique.

Figure 4 represents the F‐measure and G‐mean of different pattern recognition techniques obtained using both complete spectra and metabolite profiles as the classifier input for the original and oversampled data sets.

Oversampling the ependymoma class resulted in a higher F‐measure and G‐mean with all classification techniques when metabolite profiles were used for analysis. However, oversampling did not have a significant impact on the other two majority tumor groups. When complete spectra were used, the same pattern of increasing ependymoma classification accuracy was observed, except for AdaBoostM1‐ANN. For complete spectra, AdaBoostM1‐ANN performance peaked at 100% oversampling rate for ependymoma and 50% for the pilocytic astrocytoma group and further oversampling resulted in deterioration of AdaBoostM1‐ANN performance.

Table 3 represents the BAR, comparing the use of metabolite profile versus complete spectra as the classifier input at all bSMOTE oversampling rates. Analysis of variance showed a significant difference between classification accuracies at different oversampling rates obtained with both complete spectra and metabolite profile (*P* < 0.001).

**Table 3:** BAR of the pattern Recognition Techniques Obtained Using Metabolite Profiles and Complete Spectra as the Classifier Input at all bSMOTE Oversampling Rates.

	Original	0.5	1	1.5	2
AdaBoostM1‐NB					
Spectra	0.56	0.82	0.80	0.82	0.80
Metabolite	0.70	0.76	0.82	0.90	0.89
AdaBoostM1‐SVM					
Spectra	0.67	0.83	0.87	0.90	0.86
Metabolite	0.76	0.78	0.88	0.90	0.93
AdaBoostM1‐NN					
Spectra	0.63	0.84	0.79	0.83	0.82
Metabolite	0.78	0.83	0.93	0.92	0.92
AdaBoostM1‐LDA					
Spectra	0.58	0.70	0.76	0.80	0.80
Metabolite	0.82	0.79	0.86	0.93	0.91
Random forests					
Spectra	0.73	0.79	0.75	0.84	0.80
Metabolite	0.72	0.76	0.82	0.85	0.90

Moreover, analysis of variance performed on ependymoma class F‐measures at all oversampling rates revealed a significant difference for both complete spectra (*P* < 0.01) and metabolite profile (*P* < 0.001), demonstrating the impact of oversampling on learning algorithm performance for both input types.

**Figure 5:**
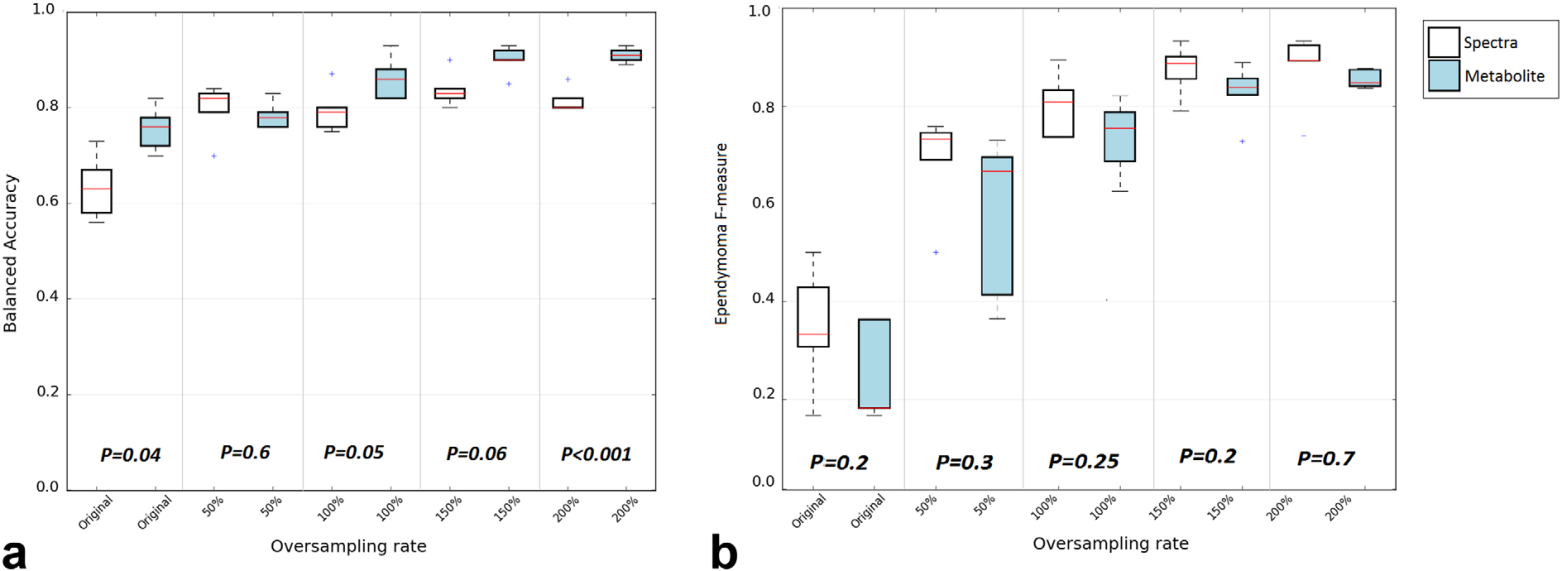
Box plots represent BARs (**a**) and ependymoma F‐measures (**b**) obtained at different sampling rates with all learning algorithms comparing complete spectra and metabolite profiles as classifier input.

Boxplots in Figure 5 represent BAR and ependymoma F‐measure obtained with all learning algorithms comparing overall classification performance for the two input features at increasing oversampling rates.

No significant difference was observed in the F‐measure between the metabolite profile and complete spectra as classifier inputs (all *P* > 0.05). An overall improvement in ependymoma F‐measure was observed as the oversampling rate of ependymoma increased (Fig. 5b). This improvement is more apparent for the complete spectra. Increasing the minority class sample size by as little as 50% increased the BAR. The increase rate was higher for spectra (26%) compared with metabolite profiles (3%).

The overall classification accuracy of learning algorithms achieved by complete spectra as input improved from 0.72 to 0.82. When metabolite profiles were used for training the learning algorithms, overall classification accuracy improved from 0.87 to 0.92.

## DISCUSSION

This study is the first to address the classification of imbalanced pediatric brain tumor MRS data. Several studies have previously classified tumor data without taking into account the data imbalance.

The cohort used in this study consisted of pilocytic astrocytoma, medulloblastoma, and ependymoma tumor types. These tumors are the three most common posterior fossa tumors in childhood but have an element of imbalance in their incidence, with ependymomas only making up about 15% of the total. These tumors have widely differing behavior, which is reflected in the treatment strategies used. For instance, ependymomas require complete resection to confer a good outcome, whereas a small residual is less important in medulloblastoma or pilocytic astrocytoma 32. Identification of tumor type prior to surgery can therefore aid surgical planning as well as allowing early organization of adjuvant treatment. Previous methods for classifying these tumors based on their MRS have shown high accuracies for the common tumors but lower accuracy for ependymomas, making this a useful test bed for imbalanced learning algorithms.

In this study, imbalance learning solutions at the data level (using the bSMOTE technique) and algorithmic level (AdaboostM1) were used to correct for this skewed distribution in the data and create robust and reliable learning algorithms.

Data created using bSMOTE were shown to have features similar to those of original ependymoma spectra and metabolite profiles. The bSMOTE ependymoma set, however, had less variability in comparison with the original data. The variation observed in the real ependymoma cohort was due to the data samples being placed away from the rest of the cohort and closer to the instances of the majority class. Depending on the minority class data distribution, bSMOTE only used safe or borderline neighbor instances of the minority class borderline samples under consideration for generating the difference feature vector and consequently new data samples. As a result, the generated data set had a lower standard deviation compared with the original data set.

Moreover, the significant differences found between the majority of the metabolites in bSMOTE ependymoma, medullobastoma, and pilocytic astrocytoma (expect for lactate and glutamine) confirmed the feasibility of using bSMOTE for generating minority class samples.

Overpopulating the ependymoma group improved the discriminatory properties of the data set. Linear projection of the data showed a better distribution and well‐defined separation of the cases with higher numbers of ependymomas (Fig. 3). A higher number of cases in the minority group also improved the impact of certain metabolites (or features) in discriminating between the groups and potentially leading into a better classification performance.

All pattern recognition techniques were shown to be more effective, because the percentage of oversampling increased for both metabolite profiles and complete spectra (Figs. 4 and 5). However, the oversampling rate required for achieving an optimum accuracy and stable classification varied depending on the choice of classifier input and learning algorithm used.

When comparing classification BAR with different rates of bSMOTE oversampled data, the best classification was achieved with AdaBoostM1‐LDA at a 150% oversampling rate (BAR = 0.93), AdaBoostM1‐SVM at a 200% oversampling rate (BAR = 0.93), and AdaBoostM1‐ANN at a 100% oversampling rate (BAR = 0.93) for metabolite profiles. It should be noted that oversampling metabolite profiles at the 150% and 200% rates allowed principal components of the data to form a linearly separable set, and as a result, LDA performed favorably in comparison with other learning algorithms.

For complete spectra, AdaBosstM1‐SVM at a 100% (BAR = 0.87) and 150% (BAR = 0.90) oversampling rate, AdaBoostM1‐NN at a 50% oversampling rate (BAR = 0.84), and random forests at a 150% oversampling rate (BAR = 0.84) were the most favorable.

It is important to note that the optimal number of trees in a random forest depends on the number of data features and available class samples. A large number of trees in a forest can cause overfitting of noisy data. Here, to obtain the optimal tree number for each oversampling rate and input type as given in Table 2, the random forest was trained using a grid of number of trees. The number of trees that maximize the F‐measure and accuracy of the classification at each sampling rate was used in developing the random forest learning algorithm.

Looking at the performance of the learning algorithms in discriminating ependymomas using metabolite profiles, AdaBoostM1‐LDA at a 150% oversampling rate was the most favorable method for classification of the minority group (F‐measure = 0.88) without jeopardizing its performance in discriminating medulloblastoma and pilocytic astrocytoma (average G‐mean = 0.94). AdaBoostM1‐SVM at 200% was the second‐best method for classification of ependymoma samples. Using the complete spectra as the training set, the best discrimination of the ependymoma group was achieved with AdaBoostM1‐SVM at 150% (F‐measure = 0.93) while maintaining the accuracy of other two groups. Except for ANN, all learning algorithms performed more favorably in discrimination of ependymoma cases with oversampled complete spectra as classifier input compared with oversampled metabolite profiles.

It should be noted that a very large oversampling rate for the minority class could turn the minority class into a new majority class and cause overfitting of the data. With complete spectra as the classification input, this was shown to be the case for random forests, AdaBoostM1‐NB, and AdaBoostM1‐SVM when the oversampling rate increased from 150% to 200%. Here, although a higher accuracy and F‐measure was obtained for the ependymoma group at the 200% oversampling rate, overfitting the data caused deterioration in classifier performance for the pilocytic astrocytoma group and resulted in a less accurate overall outcome for classifiers using complete spectra. Similarly, using complete spectra for AdaBoostM1‐ANN, the best performance was reached at 50% oversampling. In this case, an increase in the oversampling size after a certain threshold resulted in overfitting the data and threatened the outcome of classification for the majority classes. A reduction in pilocytic astrocytoma F‐measure and G mean value after a 50% oversampling rate confirmed the above finding.

It should be noted that for data sets with a huge difference between minority and majority class, oversampling the minority group at a high rate to achieve an equal balance with the majority is not necessarily the best option for solving the imbalanced learning problem. In these data sets, due to the insufficient number of minority class nearest neighbor samples, a high rate of oversampling creates a small and dense region of minority cases and results in overfitting of the data.

Comparing learning algorithms averaged BAR at each oversampling rates (Fig. 5), although higher classification accuracies were obtained using metabolite profiles as input features, no significant difference was observed between metabolite profiles and complete spectra BAR at 50%, 100% and 150% oversampling rate. The difference observed between the average BAR of the metabolite profiles and complete spectra at a 200% oversampling rate is the result of overfitting complete spectra, as discussed earlier. A significant difference however, was observed between the peak of metabolite profiles averaged BAR, achieved at 200% oversampling rate, and complete spectra averaged BAR at both 100% (*P* = 0.002) and 150% (*P* = 0.003) oversampling rate.

When examining the learning algorithms individually, different classifiers achieved comparable accuracies dependent on the used input feature and oversampling rate. AdaBoostM1‐SVM with 50% oversampled complete spectra had a BAR of 0.83—which is similar to the AdaBoostM1‐LDA BAR of 0.82—using the original cases with metabolite profiles as the input. AdaBoostM1‐SVM with complete spectra at a 100% and 150% oversampling rate was superior to AdaBoostM1‐LDA with original and 50% oversampled metabolite profiles. Similarly, random forests at a 150% oversampling rate with complete spectra had almost the same or higher accuracy compared with trained metabolite profiles at 50%, 100%, and 150% oversampling rates. It can also be argued that in the presence of a balanced training set with equally distributed data, using original complete spectra can result in a stable and accurate classification without the need for oversampling, although we were not able to test this formally.

The required size of the minority class for an optimum BAR, achieved either due to the existence of enough cases or by oversampling, depends on the number of minority and majority groups in the data set, the distribution of the data samples in each group, and the number of data features used for classification. Finally, choice of learning algorithm and classifier input is mostly dependent on data distribution, required accuracy in discriminating specific groups, and degree of postprocessing complexity.

However, as more experience is gained in applying bSMOTE to other brain tumor MRS data sets, some general rules regarding optimal oversampling and methodology will emerge. One of the motivations for comparing a wide variety of learning algorithms in this study was to give some initial insight into variation with oversampling rate and learning method. The optimal strategy found here could form an initial strategy for future studies on other data sets.

## CONCLUSION

Classification of pediatric brain tumors from MRS has many desirable characteristics. However, imbalanced distribution of the tumor classes introduces difficulty in classification and produces deterioration of the performance achieved by existing learning algorithms. Synthetic minority oversampling can overcome the rarity of data on specific tumor types.

The higher accuracy, recall, and precision obtained using the synthetic minority oversampling method demonstrates the power of the technique in discriminating the minority class and balancing the performance across different classes. These advantages should become more apparent as the number of minority class sets increases. In the presence of sufficient data samples, complete spectra achieve good accuracy and can be used for MRS pediatric brain tumor classification to reduce postprocessing complexity.

## Appendix

A summary of the AdaBoostM1 is described as follows:

Algorithm 1 Algorithm AdaBoost.M1
***Input**:* sequence of m examples $\left(x_{1},y_{1}\right),\cdots ,\left(x_{m},y_{m}\right)$ with labels $y_{i}\in Y=\left\{1,\cdots ,k\right\}$base learning algorithm **WeakLearn** integer T specifying number of iterations
***Initialize**:* $D_{1}\left(i\right)=\frac{1}{m}$ for all i.
***Do for**:* t=1,2,…,T

1. Call WeakLearn, providing it with the distribution $D_{t}$.
2. Get back a hypothesis $h_{t}:X\to Y$.
3. Calculate the error of $h_{t}:\;\epsilon_{t}=\underset{i:h_{t}\left(x_{i}\right)\neq y_{i}}{\sum }D_{t}\left(i\right)$.
  If $\epsilon_{t}>\frac{1}{2}\;$, then set T=t−1 and abort loop.
4. Set $\beta_{t}=\epsilon_{t}/\left(1-\epsilon_{t}\right)$.
5. Update distribution $D_{t:\;}D_{t+1}\left(i\right)=\frac{D_{t}\left(i\right)}{Z_{t}}\times \{\begin{array}{c} \beta_{t\;}\;if\;h_{t}\left(x_{i}\right)=y_{i}\\ 1\;otherwise \end{array}$

Where $Z_{t}$ is the normalization constant (chosen so that $D_{t+1}$ will be a distribution).
***Output**:* the final hypothesis: $h_{\text{fin}}\left(x\right)=argmax_{y\in Y}\;\underset{t:h_{t}\left(x\right)=y}{\sum }log\frac{1}{\beta_{t}}$.
